# Ciliate community structure and interactions within the planktonic food web in two alpine lakes of contrasting transparency

**DOI:** 10.1111/fwb.12828

**Published:** 2016-10-06

**Authors:** Barbara Kammerlander, Karin A. Koinig, Eugen Rott, Ruben Sommaruga, Barbara Tartarotti, Florian Trattner, Bettina Sonntag

**Affiliations:** ^1^ Ciliate Ecology and Taxonomy GroupResearch Institute for LimnologyUniversity of InnsbruckMondseeAustria; ^2^ Lake and Glacier Research GroupInstitute of EcologyUniversity of InnsbruckInnsbruckAustria; ^3^ Research Group HydrobotanyInstitute of BotanyUniversity of InnsbruckInnsbruckAustria

**Keywords:** climate change, high mountain lakes, protists, turbidity, UV radiation

## Abstract

1. Climate warming is accelerating the retreat of glaciers and recently, many ‘new’ glacial turbid lakes have been created. In the course of time, the loss of the hydrological connectivity to a glacier causes, however, changes in their water turbidity and turns these ecosystems into clear ones.
2. To understand potential differences in the food‐web structure between glacier‐fed turbid and clear alpine lakes, we sampled ciliates, phyto‐, bacterio‐ and zooplankton in one clear and one glacial turbid alpine lake, and measured key physicochemical parameters. In particular, we focused on the ciliate community and the potential drivers for their abundance distribution.
3. In both lakes, the zooplankton community was similar and dominated by the copepod *Cyclops abyssorum tatricus* and rotifers including *Polyarthra dolichoptera*,* Keratella hiemalis*,* Keratella cochlearis* and *Notholca squamula*. The phytoplankton community structure differed and it was dominated by the planktonic diatom *Fragilaria tenera* and the cryptophyte alga *Plagioselmis nannoplanctica* in the glacial turbid lake, while chrysophytes and dinoflagellates were predominant in the clear one.
4. Ciliate abundance and richness were higher in the glacial turbid lake (∼4000–27 800 Ind L^−1^, up to 29 species) than in the clear lake (∼570–7150 Ind L^−1^, up to eight species). The dominant species were *Balanion planctonicum*,* Askenasia* cf. *chlorelligera*,* Urotricha* cf. *furcata* and *Mesodinium* cf. *acarus*. The same species dominated in both lakes, except for *Mesodinium* cf. *acarus* and some particle‐associated ciliates, which occurred exclusively in the glacial turbid lake. The relative underwater solar irradiance (i.e. percentage of PAR and UVR at depth) significantly explained their abundance distribution pattern, especially in the clear water lake. In the glacial turbid lake, the abundance of the dominating ciliate taxa was mainly explained by the presence of predatory zooplankton.
5. Our results revealed an unexpected high abundance and richness of protists (algae, ciliates) in the glacial turbid lake. This type of lake likely offers more suitable environmental conditions and resource niches for protists than the clear and highly UV transparent lake.

## Introduction

Recently, molecular studies identified alpine lakes as hotspots of eukaryotic protistan diversity (Triadó‐Margarit & Casamayor, 2012; Kammerlander *et al*., 2015; Filker *et al*., 2016). However, ciliate assemblages are still an underexplored group in those lakes (Wille *et al*., 1999; Sonntag, Summerer & Sommaruga, 2011), even though within the microbial food web they are ecologically important as major link to higher trophic levels (Sommer *et al*., 2012; Lischke *et al*., 2016). Planktonic ciliate assemblages of oligo‐ and mesotrophic lowland lakes characteristically include small algivorous prostomatid and oligotrich ciliates and their community and vertical distribution patterns are related to food availability, predation and abiotic factors (Weisse & Müller, 1998; Sonntag *et al*., 2006; Van Wichelen *et al*., 2013; Posch *et al*., 2015). In the clear alpine Gossenköllesee (Austrian Central Alps), only *Balanion planctonicum*,* Urotricha* cf. *castalia* and the mixotrophic *Askenasia chlorelligera* are found during the ice‐free season, when they present a characteristic species‐specific distribution in the water column (Wille *et al*., 1999; Sonntag *et al*., 2011). Among the environmental factors known to influence the vertical distribution patterns of zooplankton, ultraviolet radiation (UVR) is important (Williamson *et al*., 2011). In clear alpine lakes, the harmful solar UV‐B radiation (280–320 nm) can easily reach the lake bottom (Laurion *et al*., 2000). In such lakes, vertical downward migrations during the day are known for phyto‐ and zooplankton (Tilzer, 1973; Tartarotti *et al*., 1999) but not for ciliates (Sonntag *et al*., 2011). For zooplankton, UVR has been identified to be an important driver in transparent lakes (Fischer *et al*., 2015), whereas high nutrient concentrations near the lake bottom can influence the vertical distribution of phytoplankton (Tilzer, 1973; Saros *et al*., 2005). Nevertheless, surface avoidance alone might be insufficient in these relatively shallow and highly UVR transparent alpine lakes. Other strategies, including the synthesis or accumulation of photoprotective compounds, or effective DNA repair mechanisms, are prerequisites and are found in a variety of planktonic organisms (Mitchell & Karentz, 1993; Sommaruga, 2001; Tartarotti *et al*., 2014).

As climate warming is accelerating glacier melting processes resulting in many newborn proglacial and ice‐contact lakes (Carrivick & Tweed, 2013; Vaughan *et al*., 2013), the high loads of inorganic particles (‘glacial flour’) in glacier‐fed lakes strongly influence abiotic factors and consequently their biota (Sommaruga, 2015). These ecosystems are light‐limited and subsequently, the primary production, the distribution of phyto‐ and zooplankton and the growth and survival of protists are affected (Hylander *et al*., 2011; Sommaruga & Kandolf, 2014; Sommaruga, 2015). Recently, next‐generation sequencing revealed high diversities of pro‐ and eukaryotes in a glacial turbid alpine lake (Kammerlander *et al*., 2015; Peter & Sommaruga, 2016). Although these lake types are in the spotlight of upcoming studies due to the strong progressive retreat of ice sheets in alpine and arctic regions (Sommaruga, 2015; Vorobyeva *et al*., 2015; Peter & Sommaruga, 2016), still virtually nothing is known about ciliates and other planktonic members in such lakes.

Here, we studied the ciliate community at the species‐level of one clear and one glacier‐fed alpine lake and the possible drivers for their species distribution against the background of potentially damaging effects of both high UVR transparency and high loads of glacial mineral particles. We expected to find different ciliate communities in respect to the prevailing environmental conditions. We sampled two adjacent alpine lakes located in the Austrian Central Alps, including one glacier‐fed turbid (Faselfadsee 3, hereafter Lake FAS 3) and one clear lake (Faselfadsee 4, hereafter Lake FAS 4), for biotic (ciliates, bacterio‐, phyto‐ and zooplankton) and physicochemical parameters on three occasions in two consecutive years.

## Methods

### Study sites

The Faselfad (FAS) lake area comprises a group of six adjacent lakes situated at altitudes between 2263 and 2620 m a.s.l. in the western Austrian Central Alps (see Figure S1 in Supporting Information). All six lakes (Lake FAS 1–6) are located below one retreating glacier, the ‘Faselfadferner’. Here, we sampled the glacial turbid Lake FAS 3 and the clear Lake FAS 4 during the ice‐free seasons on 12 July 2010 and 5 July 2011, and on 29 August 2011. Lake FAS 3 is located about 200 m below the glacier and is fed by meltwater from the glacier and runoff from the catchment, resulting in high loads of particles in the pelagial (Table 1), while Lake FAS 4 is clear and fed by seepage. Up to our knowledge, both lakes are fishless and cladoceran‐free (for more details, see Sommaruga & Kandolf, 2014; Kammerlander *et al*., 2015; Peter & Sommaruga, 2016).

**Table 1:** Geographic location, altitude, lake area, maximum depth (*Z*_max_), weather conditions, physicochemical parameters including temperature, pH, conductivity, dissolved organic carbon (DOC), dissolved phosphorus (DP), total phosphorus (TP), dissolved nitrogen (DN), optical properties such as turbidity, diffuse attenuation coefficients at 320 nm (*K*_d 320 nm_) and of photosynthetically active radiation (*K*_d PAR_), depth of 1% of surface irradiance for 320 nm UV (*Z*_1% 320 nm_) and PAR (*Z*_1% PAR_) and fraction of the water column to which 1% of the surface irradiance at 320 nm (*Z*_1% 320 nm_:*Z*_max_) and of PAR (*Z*_1% 320 nm_:*Z*_max_) penetrated and chlorophyll *a* (chl *a*) of Lakes FAS 3 (turbid) and FAS 4 (clear). Data are shown as means, minimum and maximum values. n.d., not determined. For additional parameters see Table S1 in Supporting Information

Lake	FAS 3 (turbid)	FAS 4 (clear)
Latitude Longitude	47°N 04′ 15″ 10°E 13′ 15″	47°N 04′ 27″ 10°E 13′ 34″
Altitude (m a.s.l.)	2414	2416
Lake area (km²)	0.02	0.02
*Z*_max_ (m)	17.0	15.0

Sampling date	July 2010	July 2011	August 2011	July 2010	July 2011	August 2011
Weather	Cloudy	Cloudy	Sunny	Cloudy	Cloudy	Sunny
Temperature (°C)	5.4 (3.1–7.1)	5.7 (4.6–7.2)	6.6 (5.0–8.6)	7.0 (5.8–9.3)	7.4 (5.9–8.4)	10.0 (9.1–10.6)
pH	7.5 (7.1–7.8)	7.6 (7.5–7.8)	8.0 (7.2–8.8)	7.2 (7.1–7.3)	7.4 (7.4–7.5)	7.3 (7.2–7.4)
Conductivity (μS cm^−1^)	39.7 (32.8–53.4)	37.6 (37.0–39.7)	43.2 (42.0–45.8)	51.6 (48.9–54.0)	50.6 (49.9–51.8)	49.8 (48.8–51.5)
DOC (μg L^−1^)	226.9 (182.0–282.0)	165.3 (148.0–206.0)	266.9 (220.0–318.0)	245.1 (228.0–311.0)	299.2 (245.0–364.0)	305.1 (268.0–374.0)
DP (μg L^−1^)	1.9 (1.5–2.4)	1.3 (1.0–1.9)	1.3 (1.3–1.6)	1.0 (0.8–1.1)	0.7 (0.4–0.9)	0.7 (0.6–0.9)
TP (μg L^−1^)	7.3 (5.9–9.3)	6.9 (5.0–9.1)	8.8 (7.3–10.6)	2.3 (1.8–3.4)	2.2 (1.4–4.9)	2.5 (1.7–5.7)
DN (μg L^−1^)	168 (143–184)	168 (162–178)	145 (112–199)	210 (202–230)	178 (167–183)	172 (170–176)
Turbidity (NTU)	3.2 (1.0–4.5)	4.4 (3.9–4.6)	8.6 (5.7–11.8)	0.2 (0.1–0.3)	0.2 (0.2–0.3)	0.2 (0.02–0.3)
*K*_d 320 nm_ (m^−1^)	n.d.	1.5	3.9	n.d.	0.2	0.3
*K*_d PAR_ (m^−1^)	n.d.	0.5	1.3	n.d.	0.1	0.1
*Z*_1% 320 nm_ (m)	n.d.	3.1	1.2	n.d.	21.3	15.9
*Z*_1% PAR_ (m)	n.d.	8.5	3.7	n.d.	41.6	33.1
*Z*_1% 320 nm_:*Z*_max_	n.d.	0.2	0.1	n.d.	1.4	1.1
*Z*_1% PAR_:*Z*_max_	n.d.	0.5	0.2	n.d.	2.8	2.2
Chl *a* (μg L^−1^)	3.5 (0.4–7.8)	1.3 (0.4–2.2)	8.0 (0.2–18.7)	0.8 (0.2–1.7)	0.6 (0.4–0.7)	1.9 (1.5–3.1)

### Lake sampling and *in situ* measurements

Water samples were collected with a 5‐L Schindler‐Patalas sampler from an inflatable boat above the deepest point of the lakes at 0, 1, 2, 4, 6, 8, 10, 12 and either 13 m (Lake FAS 4) or 14 m (Lake FAS 3) depth around mid‐day (within 3 h of solar noon). For ciliates, subsamples were taken (0.2–1.0 L total volume per sample and depth; triplicates) and immediately preserved with 5% Bouin's solution (final concentration). Further subsamples were fixed for phytoplankton (100 mL total volume per depth) with acidified Lugol's solution, for bacteria (50 mL total volume per depth; triplicates) with 2% formalin (final concentration) and for zooplankton (5 L per depth; triplicates). Zooplankton was concentrated on a 45‐μm mesh, transferred into 100 mL glass flasks and fixed with 4% formalin (final concentration).

In addition, living ciliates were collected by vertical net hauls (10 μm mesh size) and the water was pre‐screened through a 250‐μm net to remove large predatory zooplankton. These samples were stored in cooling boxes at *in situ* temperature.

Furthermore, subsamples were collected for the analyses of dissolved organic carbon (DOC), turbidity, water chemistry, photoprotective compounds (i.e. mycosporine‐like amino acids; MAAs) and chlorophyll *a* (chl *a*). Temperature was measured with a thermometer attached inside the water sampler. The downwelling irradiance at 305, 320, 340 and 380 nm (UVR) and between 400 and 700 nm (photosynthetically active radiation, PAR) was measured in July and August 2011 with a profiling ultraviolet radiometer (PUV‐501B, Biospherical Instruments).

### Sample processing

#### Ciliate abundance and taxonomy

Living ciliates were identified using an Olympus BX50 microscope (bright field, differential interference contrast) following the taxonomic keys of Foissner *et al*. (1991), Foissner, Berger & Kohmann (1994), Foissner *et al*. (1995), Foissner, Berger & Schaumburg (1999) and Berger (1999, 2006). Preserved samples were filtered onto cellulose nitrate filters (Sartorius, Goettingen; 0.8 μm pore size, with integrated counting grid) and stained following the quantitative protargol staining method (QPS) after Skibbe (1994) and Pfister, Sonntag & Posch (1999). From these permanent preparations, ciliates were identified and quantified under bright field (400–1000× magnification). The taxonomical assignment followed the revision of Adl *et al*. (2012).

#### Phytoplankton abundance, taxonomy and chlorophyll *a*

For qualitative and quantitative phytoplankton analysis, samples were settled in Utermöhl chambers and observed with an inverted microscope (Reichert‐Jung, Vienna) at four different magnifications (48×, 128×, 320× and 504×). For more details see Rott (1981).

For the analysis of chl *a*, 0.9–2.5 L were pre‐filtered through a 100‐μm net to remove zooplankton, concentrated on pre‐combusted glass‐fibre filters (Whatman, GF/F) and stored at −20 °C. Pigments were extracted in the dark with 90% alkaline acetone (v:v) at 4 °C for 24 h. After sonication (1 min at 35 W; Sonoplus, HD2070, Bandelin, Berlin) on ice, the extracts were cleared by filtration through glass‐fibre filters (Whatman, GF/F) and the absorbance was measured before and after acidification in a double‐beam spectrophotometer (Hitachi U‐2001, Inula, Vienna). The chl *a* concentration was calculated according to the formula of Lorenzen (1967) correcting for phaeopigments.

#### Bacterial abundance

Samples were filtered onto black polycarbonate filters (Merck Millipore GBTP, Darmstadt; 0.22 μm pore size), stained with DAPI (4′, 6′‐diamidino‐2‐phenylindole; Molecular Probes, Eugene) and semi‐automatically counted (Zeiss AxioImager; software IMI 1.5 and Zeiss Axiovision; Zeder 2005–2010; http://www.technobiology.ch). On average, ~20 images per filter were selected for counting (at least 500 cells) using a 63× objective.

#### Zooplankton abundance and taxonomy

Zooplankton was identified following the taxonomic keys of Einsle (1993), Voigt & Koste (1978a,b). The different life stages (i.e. nauplii, copepodid CI–CIII and copepodid CIV–adult females and males) of *Cyclops abyssorum tatricus* as well as the number of rotifers were counted with an inverted microscope (Leitz, Labovert) after sedimentation in Utermöhl chambers at a magnification of 100×. For biomass estimation, the *C*. *abyssorum tatricus* body length was measured at a magnification of 40× or 100×, and the body length and width of the rotifers were measured at 320×. Biomass was then calculated according to Bottrell *et al*. (1976) (rotifers) and Praptokardiyo (1979) (*C*. *abyssorum tatricus*).

### Physicochemical parameters

For dissolved organic carbon (DOC), water was stored in pre‐combusted (2 h at 500 °C) glass bottles with glass stoppers in the dark at 4 °C and subsequently filtered through pre‐combusted (2 h at 450 °C) glass‐fibre filters (Whatman GF/F, Maidstone; rinsed with 20 mL of Milli‐Q water and 10 mL of lake water). DOC was measured with a high‐temperature catalytic oxidation method (Shimadzu TOC‐V CPH, Vienna; equipped with a Shimadzu platinized‐quartz catalyst for high sensitivity analysis).

Analyses of pH, conductivity, ion concentrations and nutrients (Tables 1 and S1) were determined using standard protocols (Sommaruga‐Wögrath *et al*., 1997). Turbidity was measured using a portable turbidimeter (WTW Turb 430 T, Weilheim), which mainly detects the scattering of ‘white light’ by small particles such as glacial flour (Sommaruga & Kandolf, 2014).

### MAAs analysis

For the analysis of MAAs in the seston, 0.25–0.5 L lake water were prefiltered through a 100‐μm mesh to remove zooplankton, filtered onto pre‐combusted glass‐fibre filters (Whatman GF/F) and frozen at −80 °C. The extraction, analysis and calculation followed the protocol of Tartarotti & Sommaruga (2002).

### Statistical analyses

**Figure 1:**
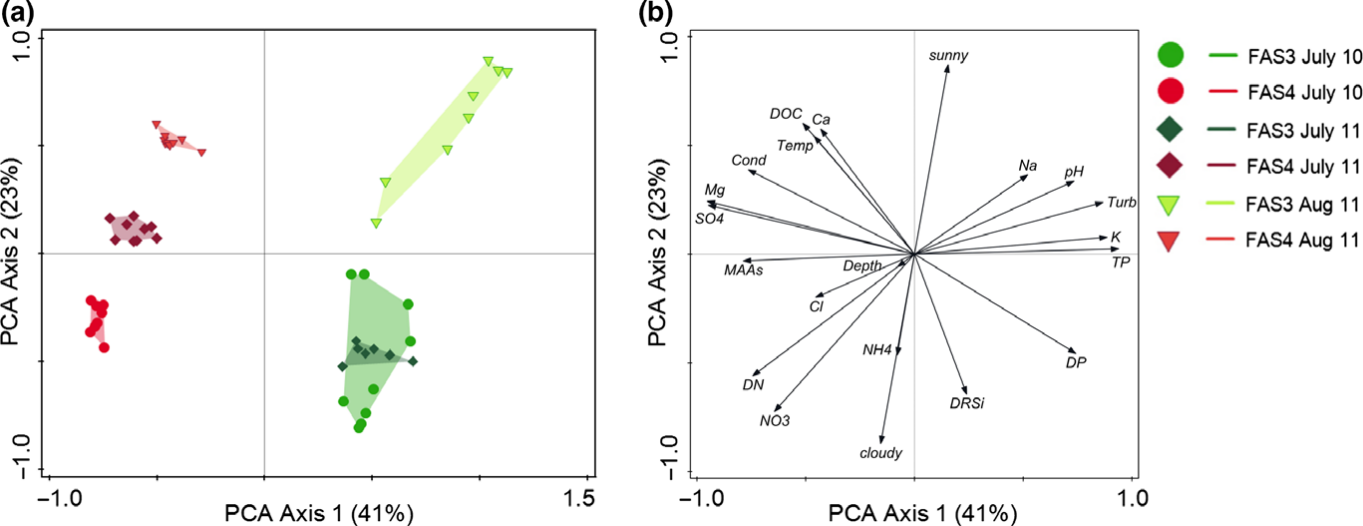
Principal component analyses (PCA) of the abiotic parameters for the glacial turbid Lake FAS 3 and the clear Lake FAS 4. Percentages indicate amount explained by the individual PCA axes. (a) Ordination of the samples; (b) ordination of the environmental parameters. Note, that when weather conditions are not included in the PCA of the abiotic parameters, the separation of the two lakes and the position of the environmental parameters remain largely the same, while the individual samples within each type of lake are ordinated closer together. Turb, turbidity; MAAs, mycosporine‐like amino acids; Cond, conductivity; Temp, temperature; for all other abbreviations see Tables 1 and S1.

**Figure 2:**
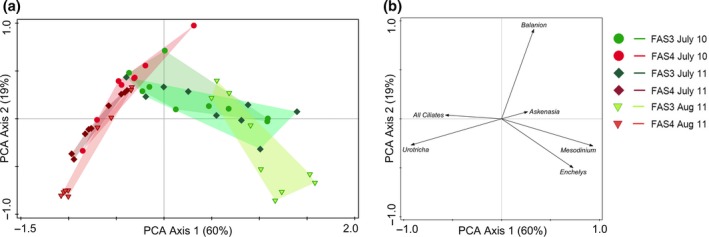
Principal component analyses (PCA) of the ciliate communities (based on abundance data) for the glacial turbid Lake FAS 3 and the clear Lake FAS 4. Percentages indicate amount explained by the individual PCA axes. (a) Ordination of the samples; (b) ordination of the species.

The diversity index for the ciliate community in each lake was calculated according to Shannon–Wiener (Mühlenberg, 1993). We used Canoco 5.04 (ter Braak & Šmilauer, 2012) in order to visualise the variation among the lakes at the three sampling events. We used principal component analyses (PCA) on independent data sets: (i) based only on the environmental data (Table 1 and S1, Fig. 1) and (ii) based solely on the ciliate community (abundance data, Fig. 2). We then used redundancy analyses (RDA) with forward selection of statistically significant parameters to analyse the variation in the ciliate species pattern in relation to the environmental parameters. The first parameter included within the forward selection process typically has the highest significant impact on the ciliate abundance distribution. Any parameter covariant with an already selected parameter will then not be available for selection within the next forward selection step (see ter Braak & Šmilauer, 2012, pages 339 ff). In our analyses, several of the explanatory variables were auto‐correlated, especially PAR and UVR (all measured wavelengths, Figure S2). Although the stepwise forward selection process allows only for one of these variables to be selected, the effect of any co‐varying parameters on the ciliate community is a combined impact and cannot be interpreted independently. In case that an alternative explanatory parameter is an equally plausible candidate for selection as major parameter, we state this in Table 2 together with the percentage this parameter would explain if chosen as the first parameter.

**Table 2:** Major drivers for the total abundance of ciliates and the dominant species (>1% of total mean abundance) assessed by RDA with forward selection of significant parameters (*P*_adj_. < 0.05). The percentage of the amount explaining the variation in the composition of the ciliate community is indicated in brackets. Note that *Mesodinium* cf. *acarus* is present only in the glacial turbid Lake FAS 3 and that the samples only for which the depth profiles of ultraviolet radiation (UVR) and photosynthetically active radiation (PAR) measurements were available (i.e. July and August 2011) were included in the analyses. See also Figure S2 with a PCA of the abiotic parameters included in these analyses

	Clear lake – FAS 4	Glacier‐fed turbid lake – FAS 3
All ciliates	Underwater solar irradiance (65% UV‐B**)†	Underwater solar irradiance (41% PAR*)
*Balanion planctonicum*	Underwater solar irradiance (67% UV‐B**)‡	*No significant parameter*
*Askenasia* cf. *chlorelligera*	Underwater solar irradiance (78% UV‐B**)	Potential predators (46% rotifers*)§
*Urotricha* cf. *furcata*	Potential predators (62% rotifers*)	*No significant parameter*
*Mesodinium* cf. *acarus*	*–*	Potential predators (78% zooplankton*)

*P* levels with Benjamini–Hochberg correction for false discovery rates during multiple testing: **<0.01, *<0.05.

Alternative factors: ^†^Zooplankton abundance (58%, **). ^‡^Zooplankton abundance (63%, **). ^§^Phytoplankton abundance (46%, **).

Redundancy analyses were chosen because of the short gradients observed in the ciliate data. The ciliate abundance data were log (*y* + 1) transformed, centred for analyses and the samples were standardised (Chord distance; Legendre & Gallagher, 2001). The arch effect in the species data remained with and without Chord or Hellinger distance transformation. Explanatory variables were centred and standardised. The RDA was then calculated for samples with PAR and UVR measurements (i.e. July and August 2011; Figure S3) with the following data: (i) for all ciliates in each lake and (ii) for the dominant ciliate groups (*B. planctonicum*,* Askenasia* cf. *chlorelligera*,* Urotricha* spp., *Mesodinium* cf. *acarus*) within each lake. The following explanatory variables were included in the analyses: depth, turbidity, physicochemical parameters (Tables 1 and S1), the relative underwater solar irradiance (i.e. % intensity of UVR at 320 nm and of PAR at the respective depths), chl *a*‐specific MAAs, abundance of bacteria, zooplankton (>1% of total abundance) including rotifers and copepods (nauplii, CI–CIII stages, CIV–adult), abundance and biovolume of phytoplankton (>5% of total biovolume) and chl *a*. Statistical significance of the explanatory parameters was assessed with 999 unrestricted Monte Carlo permutations and corrected for false discovery rate Benjamini–Hochberg correction for multiple testing implemented by Canoco 5, with a *P* < 0.05 considered as significant.

## Results

### Physicochemical parameters

**Figure 3:**
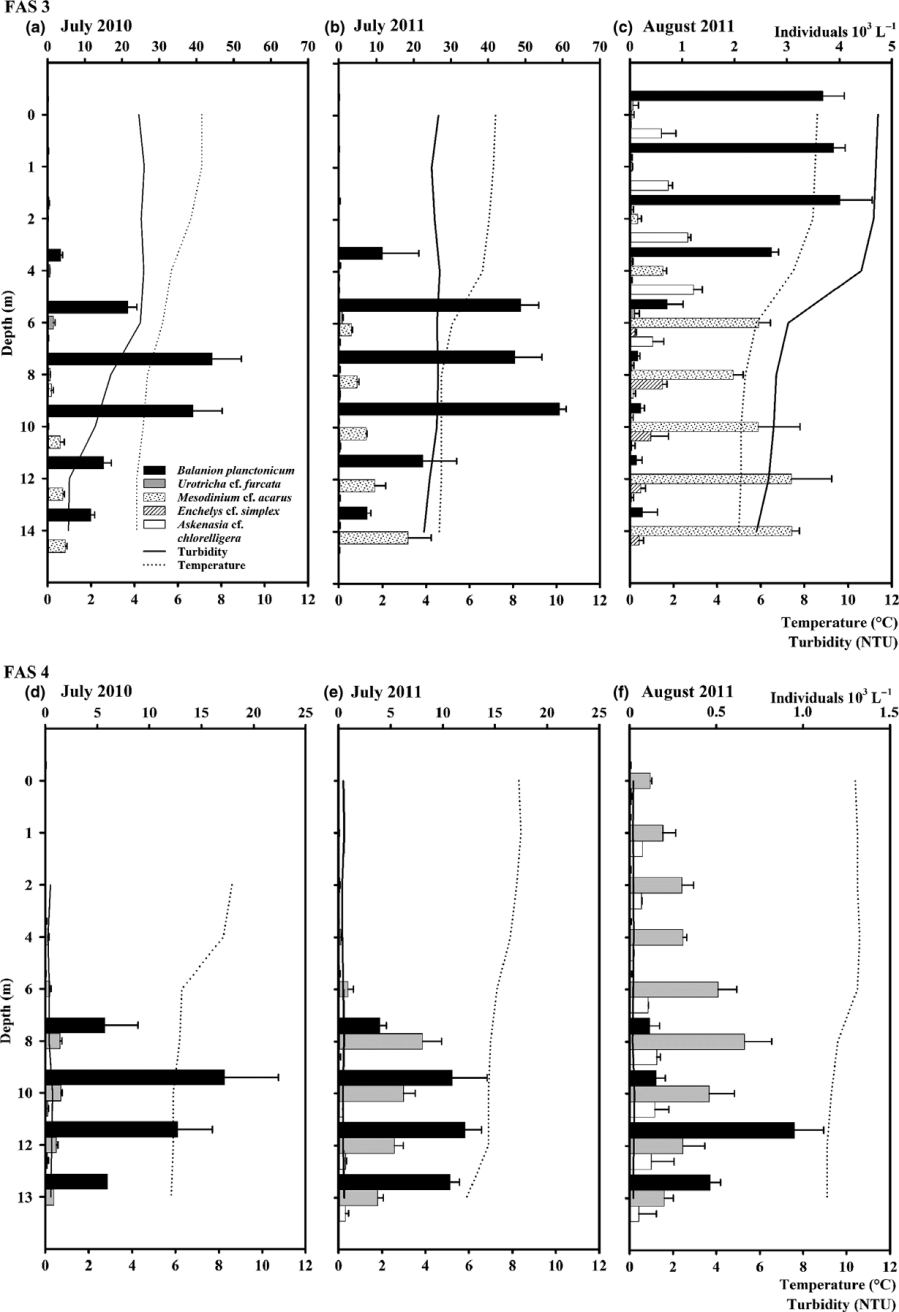
(a–f) Vertical distribution of the dominant ciliate species (>1% of the total mean abundance), turbidity and temperature in the glacial turbid Lake FAS 3 (a–c) and the clear Lake FAS 4 (d–f) during the ice‐free periods of 2010 and 2011. Error bars indicate standard deviation. Note that *Mesodinium* cf. *acarus* and *Enchelys* cf. *simplex* were exclusively found in the glacial turbid Lake FAS 3 and note the different scale bars between sampling sites and dates.

Although the lakes slightly differ in temperature (Lake FAS 3 <6 °C; Lake FAS 4 >7 °C) in both years, they showed a weak but at least temporarily stable temperature stratification with a gradual decrease in temperature near the lake bottom (Fig. 3a–f; Table 1).

The PCA of the environmental data of the FAS lakes distinctly separates the samples from the clear and the glacial turbid lake (Fig. 1a,b). The PCA axis one reflects sulphate and magnesium versus turbidity, phosphorus (total and dissolved phosphorus), potassium and pH, with sulphate and magnesium being higher in the clear and the other parameters being higher in the glacial turbid lake. We additionally calculated a PCA taking into account UVR and PAR (available only for 2011, Figure S2). UVR and PAR are scoring negatively on PCA axis one, together with sulphate and magnesium, and opposing the trends of turbidity. Thus, the first PCA axis also reflects a gradient of the intensity of downwelling irradiance. At the same time, the single sampling events (except for the July samples in the glacial turbid Lake FAS 3) are also clearly separated along the second PCA axis which is not only reflecting weather conditions (sunny versus cloudy), but also nitrate and dissolved reactive silica versus dissolved organic carbon and calcium. Still, the difference in ordination space was larger between the two lakes than among the sampling occasions.

### Ciliates

**Figure 4:**
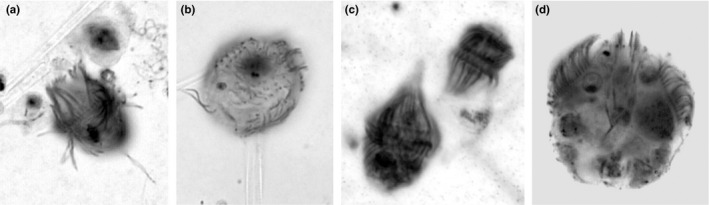
Dominant pelagic ciliates in the FAS lakes. Photographs from preserved specimens after protargol staining and taken at a magnification of 1000×. (a) *Balanion planctonicum*, (b) *Urotricha* cf. *furcata*, (c) *Mesodinium* cf. *acarus* and (d) *Askenasia* cf. *chlorelligera*.

In both lakes, the dominant ciliates were the heterotrophic prostomatids *B*. *planctonicum* (Lake FAS 3 versus Lake FAS 4: 71% versus 60% on average, considering all sampling dates) and *Urotricha* cf. *furcata* (1% versus 37%), haptorids such as *Askenasia* cf. *chlorelligera* (4% versus 3%) and *Mesodinium* cf. *acarus* (22%; present only in Lake FAS 3) (Table S2, Figs 3a–f & 4). In the glacial turbid lake, most ciliates belonged to the Prostomatea, Phyllopharyngea, Colpodea, Oligohymenophorea, Haptoria, Hypotrichia and Oligotrichia. In the clear lake, mainly Prostomatea, Haptoria and single individuals of Hypotrichia were identified (Table S2). The Shannon diversity index (*Hs*) was similar in both lakes, however, the *Hs* of the glacial turbid Lake FAS 3 (0.75) was slightly higher, though not significant, when compared with Lake FAS 4 (0.69). By contrast, species richness and the mean abundance were higher in Lake FAS 3 than in the clear Lake FAS 4 (Table S2). The PCA analysis showed clearly that the ciliate communities were different in both lakes. Based on the abundance data, the lakes were separated along the first PCA axis reflecting the presence of *Urotricha* cf. *furcata* versus *Mesodinium* cf. *acarus* and *Enchelys* cf. *simplex* (Fig. 2a,b). The latter two species were found only in the glacial turbid lake. The second PCA axis stands for the presence of *B*. *planctonicum*. In contrast to the abiotic parameters, the ciliate composition was similar within each lake at the three sampling events. For both lakes, the relative underwater irradiance (i.e. % of UV‐B and PAR at depth) appeared to be a major explanatory variable for the overall ciliate abundance pattern (Table 2). This was particularly the case in the clear lake, where the pattern of the abundant species was significantly explained by the UV‐B irradiance at depth, such as for *B*. *planctonicum* and *Askenasia* cf. *chlorelligera*. However, the abundance of potential predators (e.g. rotifers, copepods) significantly explained the abundance distribution of *Askenasia* cf. *chlorelligera* and *Mesodinium* cf. *acarus* (present only in the glacial turbid lake FAS 3) in the glacial turbid Lake, but also of *Urotricha* cf. *furcata* and *B*. *planctonicum* in the clear lake (Table 2).

The vertical distribution pattern and the number of species were similar in both July profiles in Lake FAS 3 (Fig. 3a,b; Table S2). *Balanion planctonicum* dominated between 8 and 10 m depth, *Mesodinium* cf. *acarus* at 14 m and *Urotricha* cf. *furcata* between 0 and 8 m. In contrast, we observed less species in lower abundance in the August 2011 profiles (Fig. 3c). Concurrently, *B*. *planctonicum* and *Askenasia* cf. *chlorelligera* both occurred in the upper water layers, *Enchelys* cf. *simplex* between 8 and 10 m depth, while *Mesodinum* cf. *acarus* was evenly distributed along the water column. The vertical ciliate distribution within Lake FAS 4 was similar at all sampling dates with the dominant species found between 8 and 13 m depth. The total mean abundance was similar in July 2010 and 2011, but lower in August 2011 (Fig. 3d–f; Table S2).

### Zooplankton

**Figure 5:**
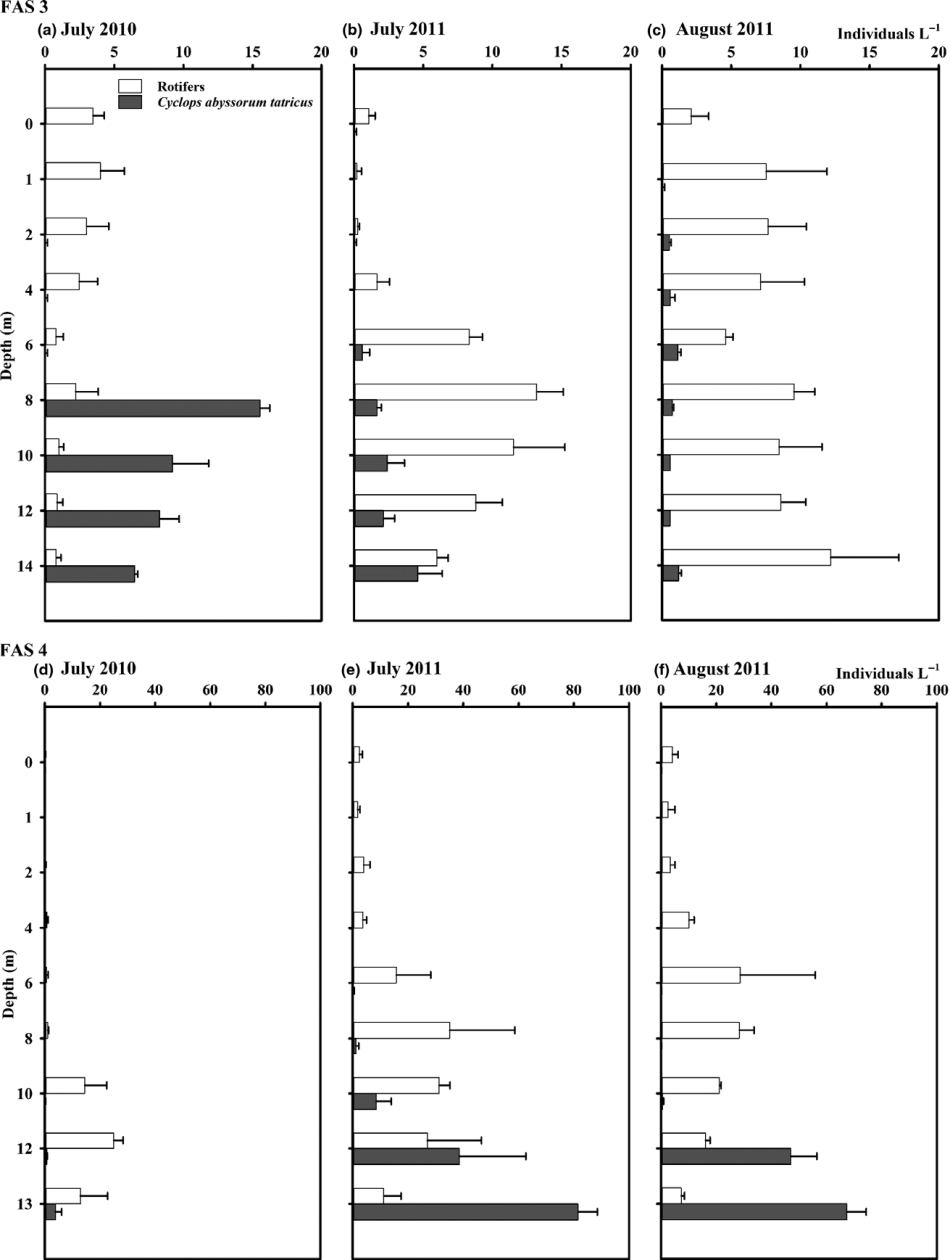
(a–f) Vertical distribution of zooplankton (rotifers and the copepod *Cyclops abyssorum tatricus*) in the glacial turbid Lake FAS 3 (a–c) and in the clear Lake FAS 4 (d–f) during the ice‐free periods of 2010 and 2011. Error bars indicate standard deviation and note the different scale bars between sampling sites.

In both lakes, we found one copepod species, *Cyclops abyssorum tatricus*, and the rotifers *Polyarthra dolichoptera*,* Keratella hiemalis*,* Keratella cochlearis* and *Notholca squamula* were dominant (Table S3; Fig. 5a–f). The zooplankton community in Lake FAS 3 consisted mainly of copepods (67% of the relative abundance and 99% of the relative biomass of zooplankton) in July 2010, while rotifers made up 82–93% of the relative zooplankton abundance, but only 8–16% of the zooplankton biomass in July and August 2011. The relative abundance of the different copepod life stages was dominated by copepodid CIV–adult life stages (48% of the relative *C. abyssorum tatricus* abundance and 80% of the relative biomass respectively) in July 2010, while nauplii dominated (69% of the relative abundance and 21% of the relative biomass of *C*. *abyssorum tatricus*) in July 2011. The copepodid CI–CIII life stages contributed with ~15% to the relative abundance of *C*. *abyssorum tatricus* (14–25% of the relative biomass) in all three sampling events. The rotifer community was composed of *P*. *dolichoptera* (22–69% of the relative abundance of rotifers and 24–73% of the relative biomass respectively), *K. hiemalis* (5–40% and 4–53%), *Kellikottia longispina* (1–7% and 2%) and *K. cochlearis* (1–4% and <1%). *Synchaeta* spp. (1–9% and up 17%) and *Filinia longiseta* (~2% and 1%) were only abundant in July 2010, *N*. *squamula* in July and August 2011 (26–40% and 9–15%) and *Ascomorpha* sp. (1% and <1%) in August 2011. The highest rotifer abundance was found between 0 and 4 m in July 2010 and between 6 and 12 m in 2011, whereas the copepods were mainly found at greater depths (8–14 m) (Fig. 5a,b). In August 2011, the zooplankton was more homogenously distributed over the whole water column (Fig. 5c).

In Lake FAS 4, the rotifers made up 92% of the relative zooplankton abundance (10% of the relative zooplankton biomass) in July 2010, while the copepods contributed with 46–50% to the zooplankton abundance and up 98% of the zooplankton biomass in 2011 (Table S3; Fig. 5d–f). Copepodid CIV–adults were the most abundant life stages in July 2010 (50% of the relative abundance and 84% of the relative biomass of *C. abyssorum tatricus*), however, nauplii dominated in July 2011 (73% of the relative abundance of *C. abyssorum tatricus* and 28% of the relative biomass respectively) and the copepodid stages CI–CIII in August 2011 (61% of the relative abundance and biomass of *C. abyssorum tatricus*). *Polyarthra dolichoptera* dominated the rotifer community (94–97% of the relative abundance and up 99% of the relative biomass of rotifers) in this clear lake. Depending on the sampling date, *K*. *hiemalis*,* K*. *cochlearis*,* K*. *longispina* and *N*. *squamula* contributed between 2% and 6% to the relative abundance and biomass of rotifers. The copepods were mainly distributed close to the bottom of the lake at all sampling occasions, also the highest rotifer abundance was found near the lake bottom in July 2010 (Fig. 5d), but in 2011 most of the rotifers were distributed between 6 and 10 m (Fig. 5e,f).

### Phytoplankton

**Figure 6:**
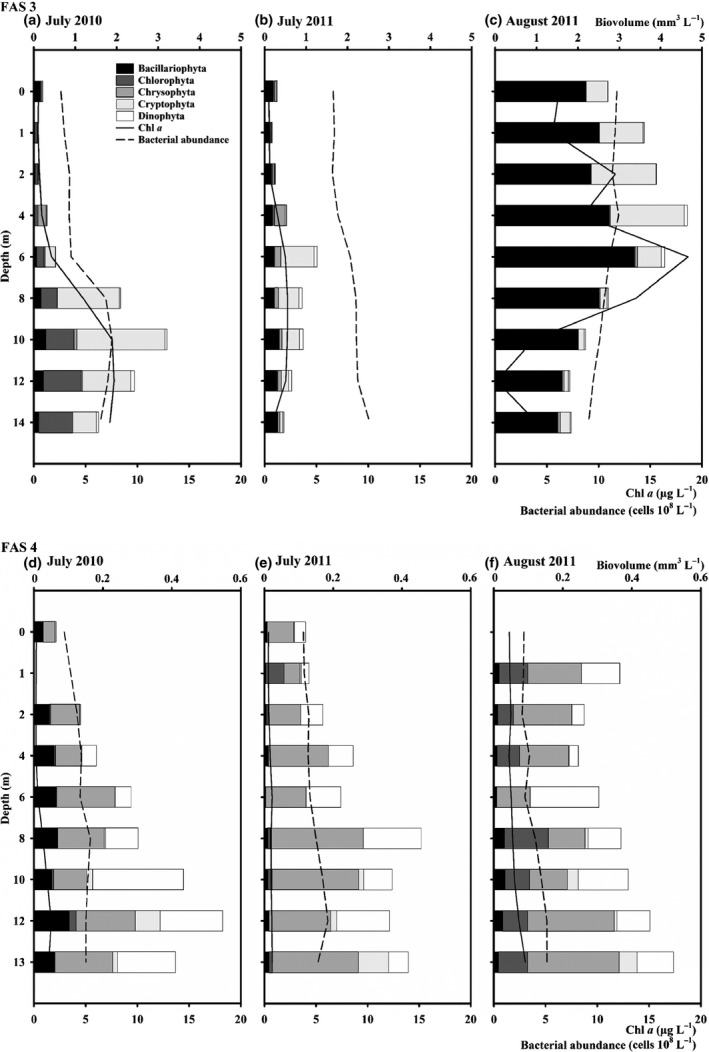
(a–f) Vertical distribution of the phytoplankton (biovolume of Bacillario‐, Chloro‐, Chryso‐, Crypto‐ and Dinophyta), chlorophyll *a* (chl *a*) and bacterial abundance in the glacial turbid Lake FAS 3 (a–c) and the clear Lake FAS 4 (d–f) during the ice‐free periods of 2010 and 2011. Note the different scale bars between sampling sites.

In both lakes, the phytoplankton community differed considerably in respect to quantities and species composition with higher values in the glacial turbid Lake FAS 3. In this lake, the phytoplankton community was dominated by diatoms (Bacillariophyceae, 10–76% of total biovolume), cryptophytes (22–53% of total biovolume) and chlorophytes (0.1–30% of total biovolumes; Fig. 6a–c; Table S4; Figure S4a–c). The highest quantities of phytoplankton were found in August 2011, when diatoms dominated and reached a peak biovolume of >4 mm³ L^−1^ (Table S4). In July 2010, the cryptophycean flagellate *Plagioselmis nannoplanctica* was dominant (53% of total biovolume) in the lower part of the water column, along with the needle‐shaped *Koliella* sp. (Chlorophyta) known from snow habitats which contributed up to 30%. The relative dominance of the planktonic diatom *Fragilaria tenera* (Bacillariophyceae) was lowest in July 2010 and highest in August 2011 (10–76% of total biovolume).

In all samples of Lake FAS 4, mainly flagellated chrysophytes (29–44% of total biovolume), dinoflagellates (28–34% of total biovolume) and the diatom *F*. *tenera* (5–20% of total biovolume) were common (Fig. 6d–f; Table S4; Fig. S4d–f). The cryptophyte *Plagioselmis nannoplanctica* contributed with only ∼5% to the total biovolume, whereas the coccal green alga *Coenococcus* sp. reached up to 19% in August 2011.

Overall, the phytoplankton biovolume and abundance depth distribution was closely related to chl *a* values in both lakes and at all sampling occasions (Fig. 6 and Figure S4). In both lakes, we observed a strong inverse depth distribution of chl *a* and phytoplankton with highest values in the lower water column, especially in July 2010 and 2011 (Fig. 6 and Figure S4). Only in the glacial turbid Lake FAS 3, we observed higher values of both chl *a* and phytoplankton biovolume in the upper water column in August 2011 (Figs 6c & S4c).

### Bacteria

In Lake FAS 3, the bacterial abundance ranged from 0.5 to 1.1 × 10^6^ cells mL^−1^ (Fig. 6a–c). In July 2010 and 2011, the bacterial abundance peaked near the lake bottom (10–14 m; Fig. 6a,b), while the highest abundance was found in the upper 0–4 m in August 2011 (Fig. 6c). In Lake FAS 4, the vertical distribution of bacteria showed a clear pattern with the lowest abundance found in the upper water layers and the highest in the deeper zone between 8 and 13 m with 3.8–4.7 × 10^5^ cells mL^−1^ (Fig. 6d–f).

## Discussion

Recent morphological and molecular studies have shown that glacial systems harbour a high microbial diversity including particle‐associated ciliate groups among others (Anesio & Laybourn‐Parry, 2012; Mieczan *et al*., 2013; Lazzaro, Risse‐Buhl & Brankatschk, 2015; Lutz *et al*., 2015). In our studied lakes, the species richness is significantly higher in the glacial turbid Lake FAS 3 than in the clear Lake FAS 4. Mainly particle‐associated ciliates contributed to the species richness in the still glacially influenced turbid Lake FAS 3. Such turbid lakes underlie glacial meltwater fluctuations, several mixing events and episodically high loads of glacial particles (Sommaruga, 2015). During glacial melting periods, glacier streams and rain events ‘wash out’ the glacier forefield and may thus introduce diverse microorganisms into a lake. The latter process also influences clear lakes, though particle‐associated protists likely do not find optimal conditions for attachment in the pelagic region. This may explain why particle‐associated bacterivorous and detritus‐feeding ciliate species were only present in the glacial turbid Lake FAS 3 (Table S2). Omnivory and life strategies, including cyst formation, probably enable these species to survive under disadvantageous conditions (Foissner *et al*., 1991, 1994; Berger, 1999, 2006; Foissner, 2008). We therefore argue that glacial turbid lakes offer suitable environmental conditions and resource niches for certain specialised protists that probably are excluded when this type of lake turns into a clear one.

In both lakes, the total ciliate abundance distribution pattern was significantly explained by the relative underwater solar irradiance (Table 2). In the clear Lake (FAS 4), the major factor explaining the distribution patterns of the dominant species was also the relative underwater solar irradiance, but this was not the case in the glacial turbid Lake (FAS 3) where zooplankton predation was more important (Table 2). When excluding the relative underwater solar irradiance from the redundancy analyses, phytoplankton abundance was the main explanatory variable for the total abundance of ciliates (31%, *P* < 0.01) in the glacial turbid Lake FAS 3. This makes sense since the dominant species found in this lake are known to be algivorous. Suitable food algae for most ciliates are unicellular chryso‐ and cryptophytes in a size range of 2–15 μm (Sonntag *et al*., 2006) such as *Plagioselmis nannoplanctica* that was highly abundant in the glacial turbid, but not in the clear lake (Fig. 6 and Figure S4, Table S4). In the glacial turbid Lake FAS 3, the abundance (and biomass) of phytoplankton, as well as the chl *a* concentration, were higher than in Lake FAS 4, especially in August 2011 (Fig. 6 and Figure S4, Table 1). Such high chl *a* peaks are not unusual in glacially influenced alpine lakes (Tolotti & Cantonati, 2000). Glacial meltwater can be a source of nutrients, especially phosphorus adsorbed to silt particles. Although only a small fraction of the phosphorus seems to be bio‐available, this input may trigger periods of production in glacier‐fed turbid lakes (Hodson, 2006; Mindl *et al*., 2007; Hodson *et al*., 2008; Sommaruga, 2015). Certain phytoplankton groups such as cryptophytes are well known to be adapted to low‐light conditions because they possess a unique light‐harvesting pigment complex in their photosynthetic apparatus (Gantt, Edwards & Provasoli, 1971; Spear‐Bernstein & Miller, 1989; Grossman *et al*., 1993; Gervais, 1997) and also, Teubner *et al*. (2001) observed higher chl *a* concentrations in a smaller size fraction of algae (<10 μm) at low‐light intensities. Interestingly, the cold‐ and low‐light‐adapted green alga *Koliella* sp. (found only in the glacial turbid Lake FAS 3; Table S4) is equipped with a highly efficient photosystem that quickly reacts to light fluctuations (La Rocca *et al*., 2015). This effective adaptation could be advantageous in light‐limited lakes such as the glacial turbid Lake FAS 3.

In both FAS lakes, the ciliate community was dominated by the common euplanktonic heterotrophic ciliates *B*. *planctonicum* and *Urotricha* spp. (Table S2; Weisse & Müller, 1998; Wille *et al*., 1999; Sonntag *et al*., 2006, 2011). These species are known to be omnivores and to have an optimal growth strategy (i.e. K‐strategy in *B*. *planctonicum* and r‐strategy in *Urotricha* spp.; Weisse *et al*., 2001). This probably explains their success in alpine lakes, even if survival strategies during unfavourable periods such as cyst formation are unknown. *Balanion planctonicum* was highly abundant particularly in the glacial turbid Lake FAS 3 and we speculate that a higher phytoplankton abundance is responsible for this. Furthermore, this species is known to be an efficient and selective feeder on small flagellates (Weisse & Müller, 1998). The different depth preferences of both prostomatid species (Fig. 3a–f) suggest an interspecific interaction probably competition for food, as found in other alpine lakes (Sonntag *et al*., 2011).

As mentioned above, in the clear lake, the abundance pattern of *B*. *planctonicum* and the mixotrophic *Askenasia* cf. *chlorelligera* were significantly explained by the relative underwater solar UVR (Table 2). *Balanion planctonicum* is sensitive to surface UVR (Sonntag *et al*., 2011), which may explain its preference for deeper water layers (Fig. 3d–f). Mixotrophic ciliates such as *Askenasia* cf. *chlorelligera* can accumulate sunscreen compounds known as mycosporine‐like amino acids (MAAs) that are crucial for survival under high levels of UVR (Sonntag, Summerer & Sommaruga, 2007; Summerer, Sonntag & Sommaruga, 2008). Although we were not able to measure MAAs in this species, MAA concentrations in seston were significantly higher in FAS 4 than in FAS 3 reflecting the different degree of UV stress for planktonic organisms in these systems (Tartarotti *et al*., 2014). In our study, the abundance of ciliate species bearing algal symbionts and described or known as mixotrophic was higher in the glacial turbid than in the clear lake (Table S2). The reduced UV stress and the capability to switch from auto‐ to heterotrophy may be advantageous in glacial turbid, but at the same time light‐limited habitats (Hinder *et al*., 1999; Sommaruga, 2015).

The abundance of predatory zooplankton significantly explained the abundance pattern of *Mesodinium* cf. *acarus* and *Askenasia* cf. *chlorelligera* in the glacial turbid lake, and of *Urotricha* cf. *furcata* and *B*. *planctonicum* in the clear lake (Table 2). The copepod *C*. *abyssorum tatricus* and rotifers such as *P. dolichoptera* and *Keratella* spp. are commonly found in Alpine lakes (Table S3; Tartarotti *et al*., 1999; Obertegger, Flaim & Sommaruga, 2008; Tiberti *et al*., 2013). Beside algae, ciliates are also in their food spectrum (Arndt, 1993; Wickham, 1995; Weisse & Frahm, 2002). However, ciliates are equipped with several defensive mechanisms/strategies, such as changing their morphology by sensing chemical cues (kairomones), repel an aggressor with extrusomes or performing escape jumps (Weisse & Sonntag, 2016 and references therein). For example, prostomatid and haptorid ciliates have characteristic swimming behaviour including spinning and jumping plus a motionless moment allowing them not only to prey efficiently, but also to avoid zooplankton predation (Foissner *et al*., 1999 and references therein; Jakobsen, Everett & Strom, 2006). Predatory zooplankton react on moving prey (e.g. Bruno, Andersen & Klørboe, 2012) and typical swimming patterns as mentioned above could be seen as an effective escape strategy (Wickham, 1995; Weisse & Frahm, 2002; Weisse & Sonntag, 2016).

In conclusion, our study unveiled an unexpected highly diverse planktonic protistan community in a glacier‐fed turbid lake. Adaptive life strategies and specific traits seem to be a prerequisite for them to thrive in these extreme habitats. Therefore, it is important to include species‐specific traits, as well as food and predator–prey interactions in future studies.

## Supporting information


**Table S1.** Additional physicochemical parameters of Lakes FAS 3 (turbid) and FAS 4 (clear).
**Table S2.** Species list of the ciliate community from the Lakes FAS 3 (turbid) and FAS 4 (clear).
**Table S3.** Species list of the zooplankton community from the Lakes FAS 3 (turbid) and FAS 4 (clear).
**Table S4.** Species list of the phytoplankton community from the Lakes FAS 3 (turbid) and FAS 4 (clear).
**Figure S1.** The study site Faselfad (FAS): The glacier‐fed turbid Lake FAS 3 and the clear Lake FAS 4 are located at ~ 2416 m a.s.l. Photo: B. Kammerlander.
**Figure S2**. PCA of the environmental parameters in the glacial turbid Lake FAS 3 and the clear Lake FAS 4 during the sampling dates when changes in the underwater solar irradiance were measured (i.e. in July and August 2011). Together with the biological explanatory parameters (e.g. bacterio‐, phyto‐ and zooplankton), these data were used in the redundancy analyses to explain the ciliate distribution. Note the distinct correlation of PAR and UVR. The impact of covariant environmental parameters on the ciliates is a combined effect and cannot be separated within the redundancy analyses (RDA). The singular, independent effect of each of these strongly auto‐correlated variables on the distribution of the ciliates is marginal.
**Figure S3**. Depth profiles of ultraviolet radiation (UVR) at 305, 320, 340, 380 nm, and of photosynthetically active radiation (PAR) in the glacial turbid Lake FAS 3 (a, b) and in the clear Lake FAS 4 (c, d).
**Figure S4.** Vertical distribution of the phytoplankton community (abundance data of Bacillario‐, Chloro‐, Chryso‐, Crypto‐ and Dinophyta), chlorophyll *a* (chl *a*) and bacterial abundance in the glacial turbid Lake FAS 3 (a–c) and the clear Lake FAS 4 (d–f) during the ice‐free periods of 2010 and 2011. Note the different scale bars between sampling sites.
