# Supplementary material for: Ciliate community structure and interactions within the planktonic food web in two alpine lakes of contrasting transparency
Source: Freshw Biol. 2016 Oct 6;61(11):1950–65. doi: 10.1111/fwb.12828 (PMC5082529; doi:10.1111/fwb.12828)
Supplement: Supplementary file 1 — Table S1. Additional physicochemical parameters of Lakes FAS 3 (turbid) and FAS 4 (clear). Table S2. Species list of the ciliate community from the Lakes FAS 3 (turbid) and FAS 4 (clear). Table S3. Species list of the zooplankton community from the Lakes FAS 3 (turbid) and FAS 4 (clear). Table S4. Species list of the phytoplankton community from the Lakes FAS 3 (turbid) and FAS 4 (clear). Figure S1. The study site Faselfad (FAS): The glacier‐fed turbid Lake FAS 3 and the clear Lake FAS 4 are located at ~ 2416 m a.s.l. Photo: B. Kammerlander. Figure S2. PCA of the environmental parameters in the glacial turbid Lake FAS 3 and the clear Lake FAS 4 during the sampling dates when changes in the underwater solar irradiance were measured (i.e. in July and August 2011). Together with the biological explanatory parameters (e.g. bacterio‐, phyto‐ and zooplankton), these data were used in the redundancy analyses to explain the ciliate distribution. Note the distinct correlation of PAR and UVR. The impact of covariant environmental parameters on the ciliates is a combined effect and cannot be separated within the redundancy analyses (RDA). The singular, independent effect of each of these strongly auto‐correlated variables on the distribution of the ciliates is marginal. Figure S3. Depth profiles of ultraviolet radiation (UVR) at 305, 320, 340, 380 nm, and of photosynthetically active radiation (PAR) in the glacial turbid Lake FAS 3 (a, b) and in the clear Lake FAS 4 (c, d). Figure S4. Vertical distribution of the phytoplankton community (abundance data of Bacillario‐, Chloro‐, Chryso‐, Crypto‐ and Dinophyta), chlorophyll a (chl a) and bacterial abundance in the glacial turbid Lake FAS 3 (a–c) and the clear Lake FAS 4 (d–f) during the ice‐free periods of 2010 and 2011. Note the different scale bars between sampling sites. [file FWB-61-1950-s001.docx]

**Supporting Information**

Table S1. Additional physicochemical parameters including ammonium (NH_4_-N), nitrate (NO_3_-N), sodium (Na), potassium (K), magnesium (Mg), calcium (Ca), dissolved reactive silica (DRSi), chloride (Cl) and sulfate (SO_4_), and chl *a-*specific mycosporine-like amino acids (MAAs) of Lakes FAS 3 (turbid) and FAS 4 (clear). Results are shown as means, minimum and maximum values.

| **Lake** | **FAS 3 (turbid)** | | | **FAS 4 (clear)** | | |
| --- | --- | --- | --- | --- | --- | --- |
| **Sampling date** | **July**  **2010** | **July**  **2011** | **August**  **2011** | **July**  **2010** | **July**  **2011** | **August**  **2011** |
|  |  |  |  |  |  |  |
| **NH_4_-N**  **(µg L^-1^)** | 2.4  1.0-4.0 | 2.4  1.0-7.0 | 1.7  1.0-4.0 | 4.4  2.0-9.0 | 1.2  1.0-2.0 | 1.1  1.0-2.0 |
|  |  |  |  |  |  |  |
| **NO_3_-N**  **(µg L^-1^)** | 176.1  147.0-194.0 | 174.9  167.0-180.0 | 127.9  89.0-201.0 | 221.6  219.0-226.0 | 183.7  177.0-187.0 | 162.3  158.0-165.0 |
|  |  |  |  |  |  |  |
| **Na**  **(mg L^-1^)** | 0.4  0.3-0.6 | 0.4  0.4-0.4 | 0.5  0.5-0.5 | 0.4  0.4-0.4 | 0.4  0.4-0.4 | 0.4  0.4-0.4 |
|  |  |  |  |  |  |  |
| **K**  **(mg L^-1^)** | 0.5  0.4-0.6 | 0.5  0.5-0.5 | 0.5  0.5-0.5 | 0.4  0.3-0.4 | 0.3  0.3-0.4 | 0.4  0.3-0.4 |
|  |  |  |  |  |  |  |
| **Mg**  **(mg L^-1^)** | 0.5  0.4-0.6 | 0.4  0.4-0.5 | 0.5  0.5-0.5 | 1.0  0.9-0.1 | 1  1.0-1.0 | 0.9  0.9-1.0 |
|  |  |  |  |  |  |  |
| **Ca**  **(mg L^-1^)** | 5.9  4.9-7.9 | 6.0  5.9-6.3 | 6.8  6.6-6.9 | 6.8  6.4-7.1 | 7.0  6.9-7.1 | 6.9  6.8-7.1 |
|  |  |  |  |  |  |  |
| **DRSi**  **(mg L^-1^)** | 808  741-963 | 680  632-727 | 514  311-658 | 617  604-632 | 519  504-549 | 588  550-665 |
|  |  |  |  |  |  |  |
| **Cl**  **(mg L^-1^)** | 0.1  0.1-0.1 | 0.1  0.1-0.1 | 0.1  0.1-0.1 | 0.1  0.1-0.1 | 0.1  0.1-0.1 | 0.1  0.1-0.1 |
|  |  |  |  |  |  |  |
| **SO_4_**  **(mg L^-1^)** | 6.2  4.7-9.1 | 5.7  5.5-6.1 | 6.6  6.4-6.9 | 14.2  13.3-15.2 | 13.7  13.4-14.0 | 12.9  12.6-13.4 |
|  |  |  |  |  |  |  |
| **Chl *a*-specific** **MAAs**  **(µg MAAs µg Chl *a*^-1^)** | 0.03  0-0.1 | 0.06  0.003-0.1 | 0.03  0.004-0.2 | 1.4  0.6-1.9 | 1.6  0.2-2.8 | 0.6  0.5-1.1 |

Table S2. Species list and abundance data of ciliates from the Lakes FAS 3 (turbid) and FAS 4 (clear) identified at each sampling occasion (July 2010, July 2011, and August 2011) from quantitative protargol stained slides. Abundance data as mean values per Liter.

| **Classification** | | | | **Species name** | **Mean abundance (Ind L^-1^)** | | | | | |
| --- | --- | --- | --- | --- | --- | --- | --- | --- | --- | --- |
|  | | | |  | **FAS 3 (turbid)** | | | **FAS 4 (clear)** | | |
|  | | | |  | **July 2010** | **July 2011** | **August 2011** | **July 2010** | **July 2011** | **August 2011** |
| **Mean total abundance**  **Min**  **Max** | | | |  | 17,062  128  46,139 | 27,827  253  67,043 | 4,028  2,886  5,300 | 5,967  20  18,904 | 7,151  45  18,155 | 568  125  1,396 |
| **Number of species** | | |  | | 26 | 22 | 15 | 6 | 9 | 5 |
| **CONthreeP** | | |  | |  |  |  |  |  |  |
|  | **Colpodea** | | | |  |  |  |  |  |  |
|  | | | | *Colpoda* cf. *steinii* | - | - | - | - | <1 | - |
|  | | | | *Colpoda lucida/ reniformis* | - | <1 | - | - | - | - |
|  | | | | unidentified Colpodea | <1 | <1 | 1 | - | - | - |
|  | **Oligohymenophorea** | | | |  |  |  |  |  |  |
|  | | | | *Cinetochilum margaritaceum* | 2 | 18 | 10 | - | <1 | - |
|  | | | | *Ctedoctema* nov. sp.  (with algal symbionts) | - | 3 | 15 | - | - | - |
|  | | | | *Dexiostoma campylum* | 2 | - | - | - | - | - |
|  | | | | *Frontonia* cf. *angusta* | 1 | - | - | - | - | - |
|  | | | | *Frontonia* sp*.* | - | - | - | - | - | <1 |
|  | | | | *Glaucoma scintillans* | - | <1 | - | - | - | - |
|  | | | | *Paramecium* cf. *putrinum* | - | - | 1 | - | - | - |
|  | | | | *Uronema* cf. *acutum* | - | <1 | - | - | - | - |
|  | | | | unidentified scuticociliate | <1 | <1 | 13 | - | - | - |
|  | | | | others | 2 | - | - | - | - | <1 |
|  | | **Phyllopharyngea** | | |  |  |  |  |  |  |
|  | | | | *Chlamydonella* sp. | - | - | - | - | 2 | - |
|  | | | | *Pseudochilodonopsis* sp. | 1 | 1 | - | - | - | - |
|  | | | | *Trochilia* sp. | - | - | - | - | - | <1 |
|  | | **Prostomatea** | | |  |  |  |  |  |  |
|  | | | | *Balanion planctonicum* | 15,113 | 22,405 | 1,748 | 5,225 | 4,205 | 188 |
|  | | | | *Plagiocampa* sp. | 2 | - | - | - | - | - |
|  | | | | *Urotricha* cf. *aspheronica* | - | 2 | 1 | - | - | - |
|  | | | | *Urotricha* cf. *castalia* | 8 | 139 | 1 | - | - | - |
|  | | | | *Urotricha* cf. *furcata* | 345 | 199 | 34 | 682 | 2,747 | 340 |
|  | | | | *Urotricha* nov. sp. 1 | 12 | 9 | - | <1 | - | - |
|  | | | | *Urotricha* nov. sp. 2 | 1 | - | 2 | - | - | - |

Table S2 continued.

| **Classification** | | | **Species name** | | **Mean abundance (Ind L^-1^)** | | | | | |
| --- | --- | --- | --- | --- | --- | --- | --- | --- | --- | --- |
|  | | |  | | **FAS 3 (turbid)** | | | **FAS 4 (clear)** | | |
|  | | |  | | **July 2010** | **July 2011** | **August 2011** | **July 2010** | **July 2011** | **August 2011** |
| **Litostomatea** | | |  | |  |  |  |  |  |  |
|  | **Haptoria** | | | |  |  |  |  |  |  |
|  | | | *Askenasia* cf. *chlorelligera* | | 18 | 183 | 470 | 57 | 196 | 38 |
|  | | | *Askenasia* nov. sp. | | 2 | - | - | - | - | - |
|  | | | *Enchelys* cf. *simplex* | | 4 | 8 | 170 | - | - | - |
|  | | | *Lagynophrya* sp. | | 2 | - | 1 | - | - | - |
|  | | | *Mesodinium* cf. *acarus* | | 1,497 | 4,830 | 1,550 | - | - | - |
|  | | | *Phialina* sp. | | 1 | - | - | - | - | - |
|  | | | *Rhabdoaskenasia* sp. | | 2 | - | - | - | - | - |
|  | | | *Trachelophyllum* cf. *apiculatum* | | - | 2 | - | - | - | - |
|  | | | *Lacrymaria* sp. | | - | - | - | - | <1 | - |
|  | | | *Monodinium* sp. | | - | - | - | 2 | - | - |
|  | **others** | | | | 36 | 22 | 10 | - | <1 | - |
| **Spirotrichea** | | | |  |  |  |  |  |  |  |
|  | **Oligotrichia** | | | |  |  |  |  |  |  |
|  | | | *Rimostrombidium* sp. | | 1 | - | - | - | - | - |
|  | **Hypotrichia** | | | |  |  |  |  |  |  |
|  | | | *Anteholosticha* sp. | | 1 | - | - | - | - | - |
|  | | | *Halteria* cf. *bifurcata* | | 1 | - | - | - | - | - |
|  | | | *Urosomoida* sp. | | 1 | - | - | - | - | - |
|  | | | *Urosomoida* cf. *longa* | | - | <1 | - | - | - | - |
|  | | | *Urosomoida* cf. *pseudofurcata* | | 1 | - | - | 1 | 1 | - |
|  | | | unidentified species | | 9 | <1 | 2 | - | - | - |
|  | | **Euplotia** | | |  |  |  |  |  |  |
|  | | | *Euplotes affinis* | | - | <1 | - | - | - | - |
|  | | | *Aspidisca* sp. | | - | <1 | - | - | - | - |

Table S3. Species list of the zooplankton community from the Lakes FAS 3 (turbid) and FAS 4 (clear) at each sampling occasion (July 2010, July 2011, and August 2011) including abundance and biomass data (mean values per Liter). CI‑CIII: copepodid life stages CI-CIII, CIV-adult: copepodid life stages CIV to adults.

| **Classification** | | **Species name/  developmental stage** | **Mean abundance (Ind L^-1^)**  **Mean biomass (µg dry weight L^-1^)** | | | | | |
| --- | --- | --- | --- | --- | --- | --- | --- | --- |
|  | |  | **FAS 3 (turbid)** | | | **FAS 4 (clear)** | | |
|  | |  | **July 2010** | **July 2011** | **August 2011** | **July 2010** | **July 2011** | **August 2011** |
| **Mean total abundance** | | | 6.6 | 7.0 | 8.0 | 7.4 | 29.1 | 28.0 |
| **Mean total biomass** | | | 13.645 | 2.114 | 1.367 | 3.209 | 21.779 | 43.018 |
|  | | |  |  |  |  |  |  |
|  | **Copepoda** *Cyclops abyssorum tatricus* | | 4.4  13.551 | 1.3  1.944 | 0.6  1.153 | 0.6  2.897 | 14.4  21.133 | 12.9  42.344 |
|  | | Nauplii | 1.7  0.784 | 0.9  0.418 | 0.4  0.170 | 0.1  0.056 | 10.6  5.935 | 2.6  1.430 |
|  | | CI-CIII | 0.6  1.919 | 0.2  0.322 | 0.1  0.295 | 0.2  0.411 | 2.8  8.638 | 7.8  25.767 |
|  | | CIV-adult | 2.1  10.848 | 0.2  1204 | 0.1  0.688 | 0.3  2.430 | 1.0  6.560 | 2.5  15.148 |
|  | **Rotatoria** | | 2.2  0.094 | 5.7  0.171 | 7.4  0.214 | 6.8  0.312 | 14.7  0.646 | 15.1  0.674 |
|  | | *Ascomorpha* sp. | - | - | 0.1  x | - | - | - |
|  | | *Filinia longiseta* | 0.04  x | x  x | - | x  x | x  x | - |
|  | | *Kellicottia longispina* | 0.1  x | 0.4  x | 0.1  x | x  x | 0.3  x | x  x |
|  | | *Keratella cochlearis* | 0.02  x | 0.2  x | 0.3  x | -  x | 0.3  x | 0.2  x |
|  | | *Keratella hiemalis* | 0.3  x | 2.3  0.091 | 0.4  0.015 | 0.4  x | x  x | x  x |
|  | | *Notholca squamula* | 0.1  x | 1.5  x | 3.1  0.032 | x  x | x  x | 0.2  x |
|  | | *Polyarthra dolichoptera* | 1.4  x | 1.3  0.058 | 3.4  0.156 | 6.4  0.294 | 14.0  0.638 | 14.7  0.670 |
|  | | *Synchaeta* spp. | 0.2  x | x  x | x  x | x  x | x  x | - |

x= present, <1% of total abundance and biomass

Table S4. Species list of the phytoplankton community from the Lakes FAS 3 (turbid) and FAS 4 (clear) at each sampling occasion (July 2010, July 2011 and August 2011) including abundance and biovolume data (mean values per Liter).

| **Classification** | | **Species name** | **Mean abundance (Ind L^-1^) Mean biovolume (mm^3^ L^-1^)** | | | | | |
| --- | --- | --- | --- | --- | --- | --- | --- | --- |
|  | |  | **FAS 3 (turbid)** | | | **FAS 4 (clear)** | | |
|  |  | | **July 2010** | **July 2011** | **August 2011** | **July 2010** | **July 2011** | **August 2011** |
|  |  | |  |  |  |  |  |  |
| **Mean total abundance** | | | 3,748,743 | 2,906,651 | 10,278,410 | 1,172,926 | 1,608,221 | 2,252,435 |
| **Mean total biovolume** | | | 1.175 | 0.607 | 3.059 | 0.297 | 0.281 | 0.365 |
| **Bacillariophyceae** | | | 398,100 0.124 | 752,347 0.231 | 7,292,023 2.308 | 186,634 0.060 | 24,566 0.008 | 64,185 0.018 |
|  | *Cyclotella* sp. | | - | - | - | - | - | x |
|  | *Fragilaria tenera* | | 398,100 0.124 | 752,347 0.231 | 7,292,023 2.308 | 186,634 0.060 | 24,566 0.008 | 53,773 0.017 |
| **Charophyceae** | | |  |  |  |  |  |  |
|  | Conjugatophyceae indet. | | - | - | - | x | - | - |
|  | *Cosmarium* sp. | | - | - | - | x | - | - |
| **Chlorophyceae** | | | 1,139,711 0.356 | 40,815 0.020 | 11,811 0.004 | 30,445 0.004 | 185,171 0.010 | 1,029,966 0.069 |
|  | *Chlamydomonas* sp. | | x | x | x | x | - | - |
|  | *Coenococcus* sp. | | - | - | - | 4,746 0.001 | 35,891 0.006 | 1,026,992 0.068 |
|  | *Koliella* sp. | | 1,133,326 0.351 | 23,135 0.007 | 2,556 0.001 | - | - | - |
|  | *Lauterbornia* sp. | | - | - | - | x | - | - |
|  | *Oocystis* sp. | | - | - | x | x | x | x |
| **Chrysophyceae** | | | 489,121 0.042 | 1,509,003 0.090 | 1,163,977 0.028 | 827,793 0.116 | 1,219,784 0.163 | 1,047,854 0.162 |
|  | *Chrysococcus* sp. | | - | - | - | 143,119 0.027 | 65,393 0.012 | 41,772 0.016 |
|  | *Chrysosphaerella* sp. | | - | 105,481 0.037 | - | 1,426 0.001 | 1,047 0.006 | - |
|  | *Kephyrion* sp. | | - | - | - | 231,113 0.01 | 545,217 0.027 | 423,938 0.021 |
|  | *Mallomonas* sp. | | x | - | - | - | - | - |
|  | *Ochromonas* sp. | | - | - | - | - | 32,907 0.023 | - |
|  | Picoplankton  (2-3 µm) | | x | - | - | - | - | - |
|  | unidentified chrysophyte  (2-15 µm) | | 470,912 0.040 | 1,403,522 0.053 | 1,163,977 0.028 | 427,328 0.059 | 554,125 0.082 | 582,144 0.125 |
|  | *Uroglena* sp. | | - | - | - | x | x | - |
|  |  | |  |  |  |  |  |  |

Table S4 continued

| **Classification** | | **Species name** | **Mean abundance (Ind L^-1^) Mean biovolume (mm^3^ L^-1^)** | | | | | |
| --- | --- | --- | --- | --- | --- | --- | --- | --- |
|  | |  | **FAS 3 (turbid)** | | | **FAS 4 (clear)** | | |
| **Cryptophyceae** | | | 1,640,764 0.623 | 575,507 0.219 | 1,793,812 0.682 | 57,727 0.014 | 62,176 0.014 | 43,197 0.013 |
|  | *Cryptomonas* sp. | | - | **-** | **-** | x | **-** | **-** |
|  | *Plagioselmis nannoplanctica* | | 1,640,764 0.623 | 575,507 0.219 | 1,793,812 0.682 | 56,302 0.014 | 55,267 0.014 | 43,197 0.013 |
| **Cyanophyceae** | | | x | x | x | x | x | x |
| **Dinophyceae** | | | 9,646 0.028 | 23,579 0.046 | 16,777 0.038 | 31,776 0.100 | 27,987 0.084 | 44,921 0.102 |
|  | *Gymnodinium* sp. | | 1,273 0.011 | 2,001 0.017 | 989 0.008 | 4,757 0.033 | 4,002 0.034 | 6,728 0.057 |
|  | *Gyrodinium* sp. | | - | - | - | 9,223 0.028 | - | - |
|  | *Peridinium* sp. | | - | - | - | x | - | - |
|  | unidentified dinophyte | | 8,373 0.018 | 21,578 0.029 | 15,788 0.029 | 16,681 0.037 | 23,985 0.050 | 38,193 0.045 |

x= present, < 5% of total biovolume

Fig. S1


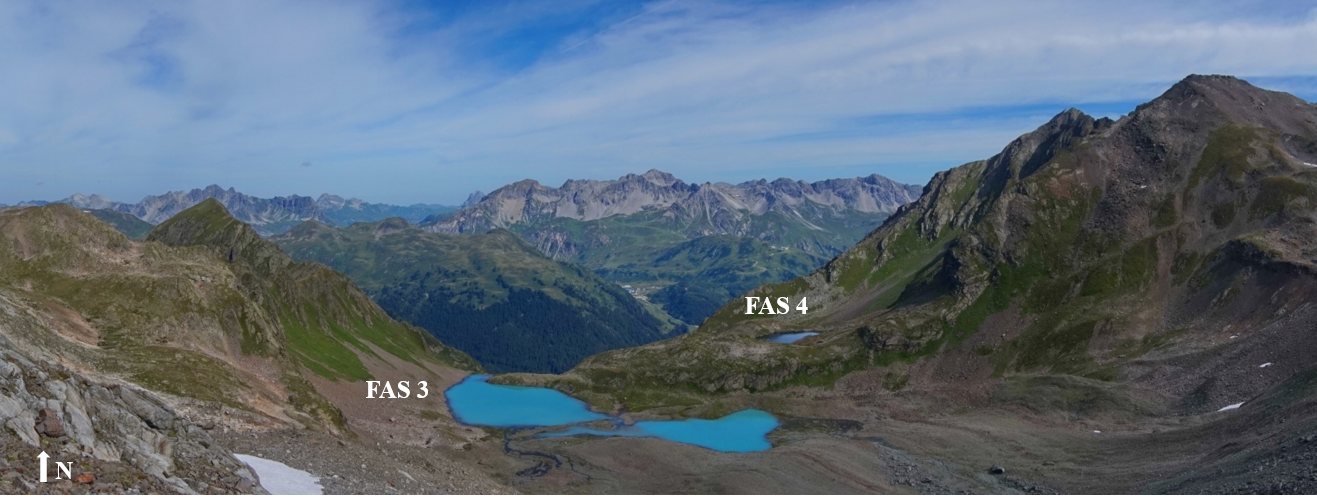


Fig. S2


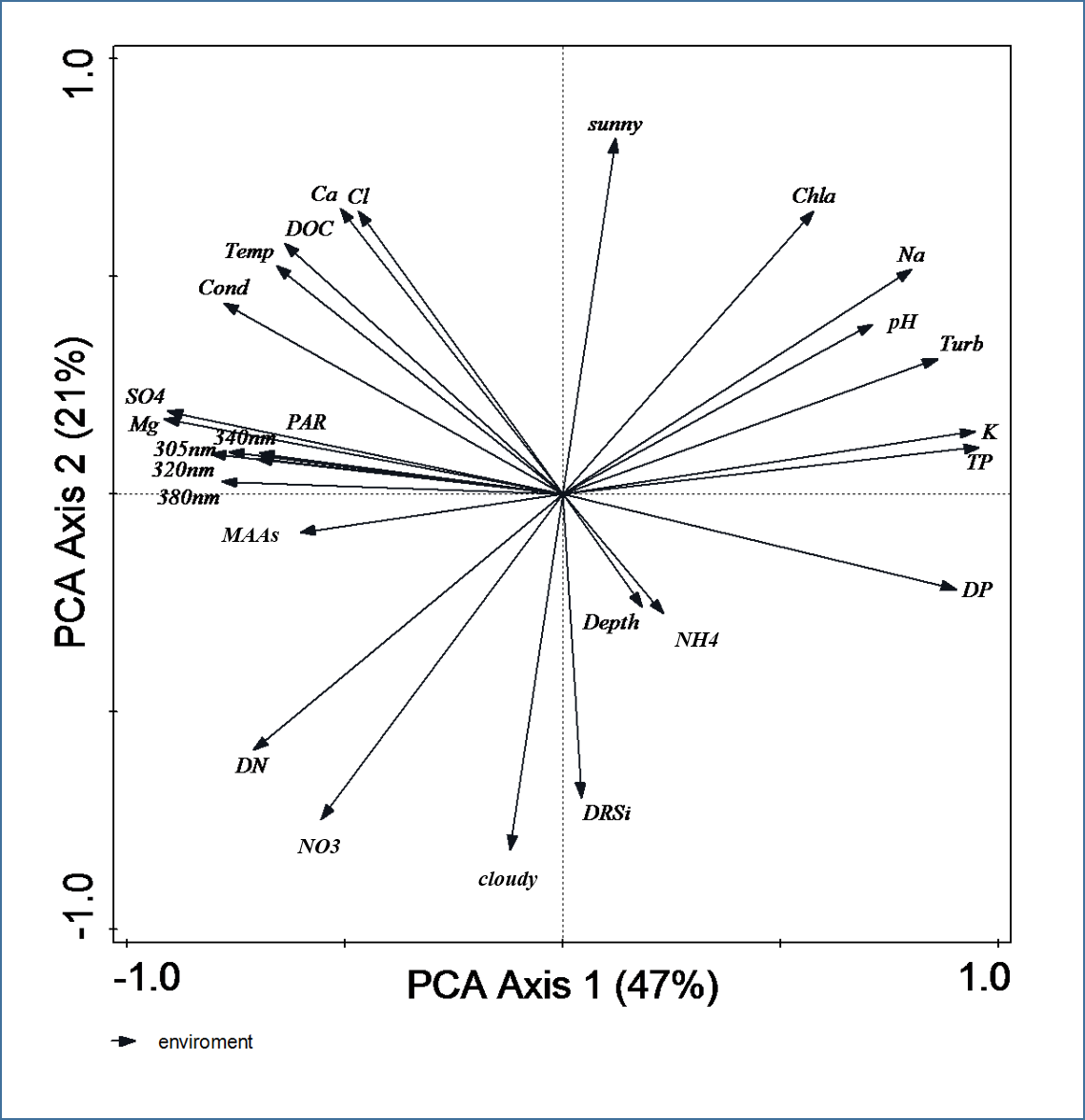


Fig. S3

Fig. S4 a-c








Fig. S4 d-f
